# Safe and Effective Analgesia Using Ultrasound-Guided Brachial Plexus Block for Upper Limb Compartment Syndrome: A Case Report

**DOI:** 10.7759/cureus.95698

**Published:** 2025-10-29

**Authors:** Danny Alexander Utreras Freire, Paola Vanessa Astudillo Ochoa, Néxar Fernando Ron Valarezo, Wilson Andrés Saraguro Armijos, Angélica María Picón Montero, Luis Arnaldo Meza Guerrero

**Affiliations:** 1 Anesthesiology, Hospital General Marco Vinicio Iza, Lago Agrio, ECU; 2 General Practice, Clíníca Pomasqui, Quito, ECU; 3 General Practice, Hospital General Marco Vinicio Iza, Lago Agrio, ECU; 4 General Practice, Jara Seguridad, Lago Agrio, ECU

**Keywords:** brachial plexus block, compartment syndrome, ultrasound-guided blocks and vascular access, upper limb, upper limb surgery

## Abstract

Upper limb compartment syndrome (ULCS) is a life-threatening and uncommon medical disorder that develops when the pressure within one or more muscle compartments of the upper limb increases. Consequently, it limits blood flow and leads to ischemia of the muscles and nerves. The objective of this case report was to analyze the safe and effective utilization of ultrasound-guided posterior interscalene brachial plexus block (USG-ISB) to document sufficient analgesia, maintenance of respiratory function, and hemodynamic stability in the treatment of ULCS. In this case report, the patient presented with severe right upper limb pain and signs of ischemia due to trauma. The primary treatment, surgical fasciotomy, was performed in an emergency setting to alleviate compartment pressure. An USG-ISB was applied as an adjunct to anesthetic management to provide safe and adequate analgesia and to stabilize the patient hemodynamically. It also reduced the need for systemic opioid use. This regional anesthesia technique was not associated with any perioperative complications, and no further morbidity was observed during the 23-day hospital stay. Following adequate regional anesthesia administration, the patient reported only mild disability (QuickDASH score of 5) at the 3-month follow-up. This case emphasizes the importance of USG-ISB as an effective and safe technique for achieving adequate analgesia and hemodynamic stabilization without delaying the diagnosis of compartment syndrome, owing to appropriate monitoring (ultrasound, serial neurovascular checks) during hospitalization and the involvement of a multidisciplinary team.

## Introduction

Upper limb compartment syndrome (ULCS) is a life-threatening and uncommon medical disorder that develops when the pressure within one or more muscle compartments of the upper limb increases. Consequently, it limits blood circulation and leads to ischemia of the muscles and nerves [1]. The trauma can result from fractures, crush injuries, or prolonged compression and requires immediate treatment before irreparable tissue damage occurs [2]. The primary, or gold standard, treatment for ULCS is surgical decompression (fasciotomy) to alleviate compartment pressure and pain, as well as to improve perfusion during the operative period, which can be particularly challenging.

Early diagnosis and intervention are crucial in the management of ULCS, since delayed treatment may result in irreversible functional impairment such as muscle necrosis and nerve damage [3]. Thus, fasciotomy is considered a life-saving or limb-saving intervention [4]. However, there remains a challenge in managing pain and optimizing perfusion during fasciotomy. Regional anesthesia is frequently utilized for pain control, but there is ongoing debate regarding its implications, as some studies have suggested that regional anesthesia may mask the cardinal symptoms of ULCS, potentially delaying diagnosis. Conversely, other studies have demonstrated that with adequate monitoring and clinical observation, including the use of ultrasound, regional anesthesia can be safely applied without delaying diagnosis or masking key symptoms. This case explores the safe and effective application of ultrasound-guided posterior interscalene brachial plexus block (USG-ISB) to achieve adequate analgesia and hemodynamic stability during fasciotomy [5-7], and demonstrates how the associated risks can be mitigated.

## Case presentation

A 43-year-old male agricultural laborer presented to the ED with severe right upper limb and right hemithorax pain accompanied by paresthesia. On presentation, the Visual Analog Scale (VAS) score was 10/10 for 24 hours’ duration, following trauma caused by the fall of several wooden boards. The nature of the trauma was a crush injury. The patient was struck by several large, heavy, wet wooden boards (weighing over 80 kg) that compressed his right upper extremity and hemithorax for approximately 10 minutes before being dislodged by bystanders. As a result of the prolonged external pressure, he developed significant soft tissue bruising, and the sustained compression likely caused microvascular damage, interstitial bleeding, and obstruction of venous outflow, leading to increased compartmental pressure and ischemia in the affected tissues. Past medical history included arterial hypertension (1 year, irregular treatment with losartan 50 mg daily) and chronic venous insufficiency of the lower limbs. No surgical history, allergies, or other significant comorbidities were reported.

Initial evaluation

**Figure 1:**
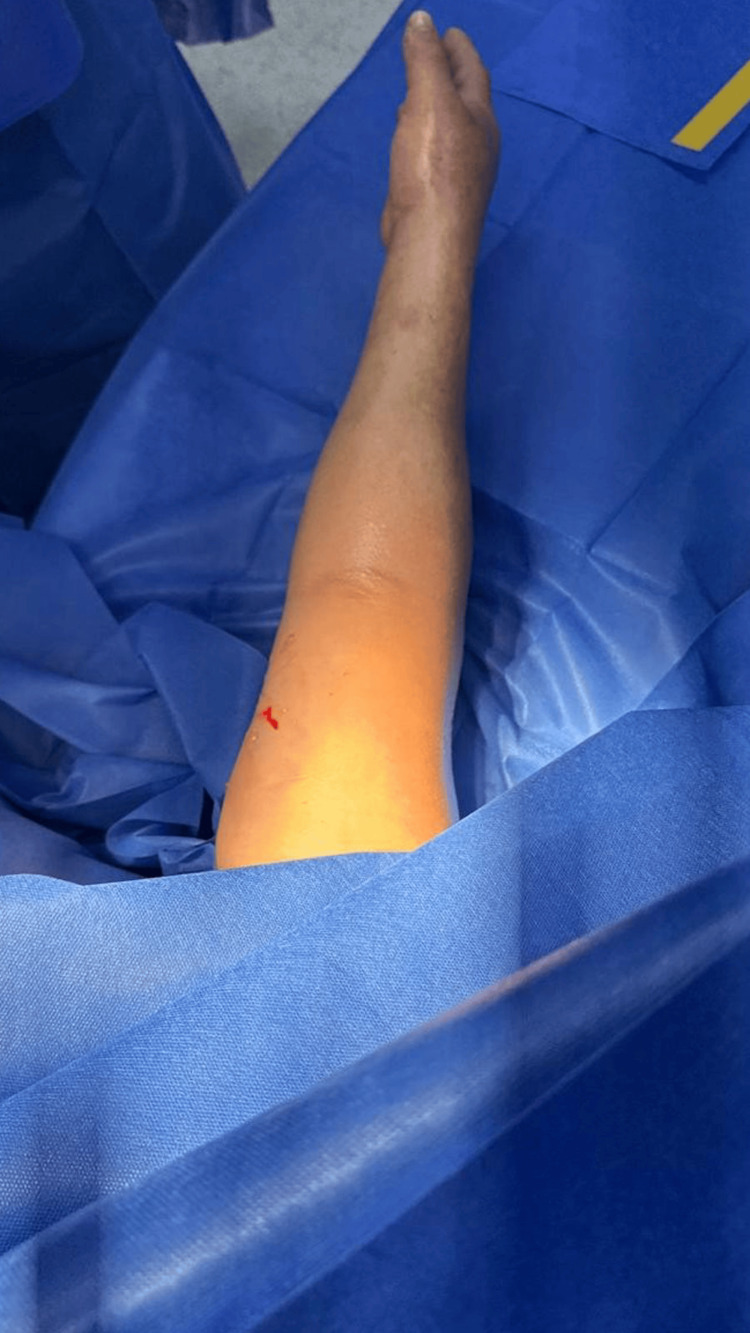
Preoperative clinical image of the right upper limb demonstrating swelling, skin tension, and early ischemic changes consistent with compartment syndrome before surgical decompression.

At admission, vital signs demonstrated elevated blood pressure (TA 130/93 mmHg), tachycardia (HR 115 bpm), respiratory rate (RR) of 20 rpm, oxygen saturation (SpO₂) of 90%, and temperature of 36.3°C. Capillary refill time in the affected limb was greater than 3 seconds, with a VAS pain score of 10/10. The patient’s baseline oxygen saturation of 90% was attributed to hypoventilation resulting from severe pain and possible minor thoracic contusion secondary to the wooden boards that struck his chest. Localized edema and basal crackles were noted on physical examination; however, no abnormalities were detected on the chest X-ray. Therefore, the patient did not have pneumothorax, hemothorax, or major lung trauma. Furthermore, as the patient’s oxygen saturation improved progressively with pain relief interventions and during the anesthetic procedure, maintaining a consistent SpO₂ of 95-97% throughout the intraoperative and postoperative periods, supplemental oxygen was not required. On examination, the patient exhibited marked edema, pallor, severe pain on palpation, reduced distal pulse, and capillary refill greater than 3 seconds. The right hemithorax showed edema, erythema, and basal crackles (Figure 1).

Diagnostic workup

**Figure 2:**
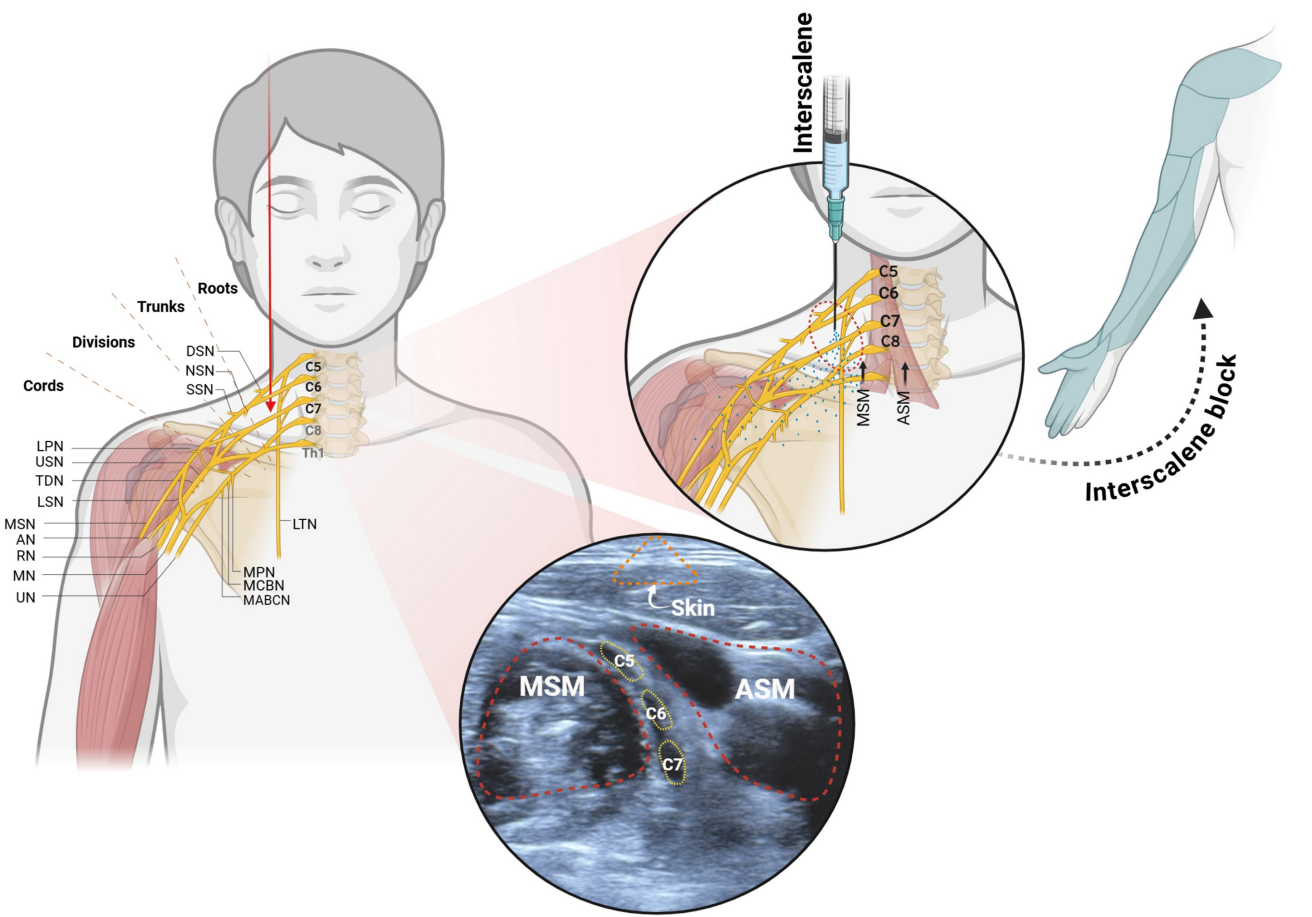
Ultrasound-guided posterior interscalene brachial plexus block technique. Created by the author Danny Alexander Utreras Freire using BioRender.com (2025). Retrieved from https://BioRender.com/m75ibue Roots: C5: Cervical root 5; C6: Cervical root 6; C7: Cervical root 7; Th1: Thoracic root 1; Th2: Thoracic root 2. Major nerves: MSN: Musculocutaneous nerve; MN: Median nerve; RN: Radial nerve; UN: Ulnar nerve; MCBN: Medial cutaneous nerve of the arm (medial brachial cutaneous); MABCN: Medial antebrachial cutaneous nerve; MPN: Medial pectoral nerve; LPN: Lateral pectoral nerve; USN: Upper subscapular nerve; LSN: Lower subscapular nerve; TDN: Thoracodorsal nerve; SSN: Suprascapular nerve; NSN: Nerve to subclavius; DSN: Dorsal scapular nerve; LTN: Long thoracic nerve; AN: Axillary nerve. Muscles: ASM: Anterior scalene muscle; MSM: Middle scalene muscle.

Chest X-ray imaging revealed no bone injury or significant pulmonary changes. Ultrasound of the right upper limb demonstrated diffuse muscle thickening, loss of normal muscle architecture, intramuscular edema, and altered vascular flow on Doppler, findings consistent with ULCS. Laboratory investigations showed a WBC count of 16.08 ×10⁹/L, neutrophils 35.8%, lymphocytes 4.2%, monocytes 0.3%, hemoglobin (Hb) 17.6 g/dL, hematocrit (Hct) 49.3%, mean corpuscular volume (MCV) 28.9 µm³, mean corpuscular hemoglobin (MCH) 31.8 pg, platelets 234,000/µL, creatinine 0.83 mg/dL, International Normalized Ratio (INR) 1.62, thromboplastin time (TTP) 29.1, and prothrombin time (TP) 12.1, indicating an inflammatory response secondary to trauma (Figure 2).

The case was managed at Hospital General Marco Vinicio Iza, a public hospital located in the Amazon region of Ecuador, where access to advanced diagnostic equipment is limited. At the time of admission, a device for measuring intracompartmental pressure was not available, and the nearest referral center with higher-level trauma care resources was located more than eight hours away by land. Given the patient’s critical presentation and the risk of irreversible ischemic damage, delaying treatment for confirmatory testing was not feasible.

The diagnosis was therefore established based on the classical clinical findings of severe pain, tense swelling, neurological impairment, and diminished distal perfusion, supported by ultrasound evidence of diffuse muscle edema and architectural disruption. These findings provided sufficient diagnostic certainty to proceed with emergency surgical decompression, which resulted in a favorable clinical recovery. In this setting, a prompt intervention approach was adopted when compartment syndrome was strongly suspected, particularly appropriate in resource-limited or remote environments where confirmatory pressure measurement is unavailable.

Considerations on differential diagnosis

In the acute setting of upper limb trauma with pain, swelling, and vascular compromise, several differential diagnoses were initially considered. Deep vein thrombosis (DVT) was ruled out based on the acute traumatic onset and the absence of prior risk factors. Doppler findings showed altered intramuscular perfusion rather than venous obstruction. Cellulitis or infectious fasciitis was considered but excluded given the lack of systemic signs of infection, normal temperature, and laboratory results without a marked inflammatory response beyond leukocytosis attributable to trauma. Fracture or dislocation was excluded by plain radiography, which revealed no bony abnormalities. Finally, isolated vascular injury was considered; however, ultrasound demonstrated diffuse muscular edema and architectural disruption, findings more consistent with compartment syndrome than with localized arterial laceration. Taken together, the combination of clinical findings, severe pain disproportionate to trauma, neurological symptoms, diminished distal pulses, and ultrasound evidence, supported the diagnosis of ULCS.

Initial management

Emergency measures included oxygen therapy, IV fluids, and analgesics. The patient underwent surgical fasciotomy and wound cleaning for decompression. Since the condition was severe and required urgent surgical intervention, anesthesia management was critical. The anesthesia team chose a regional anesthesia technique to achieve effective analgesia and reduce systemic opioid consumption.

**Figure 3:**
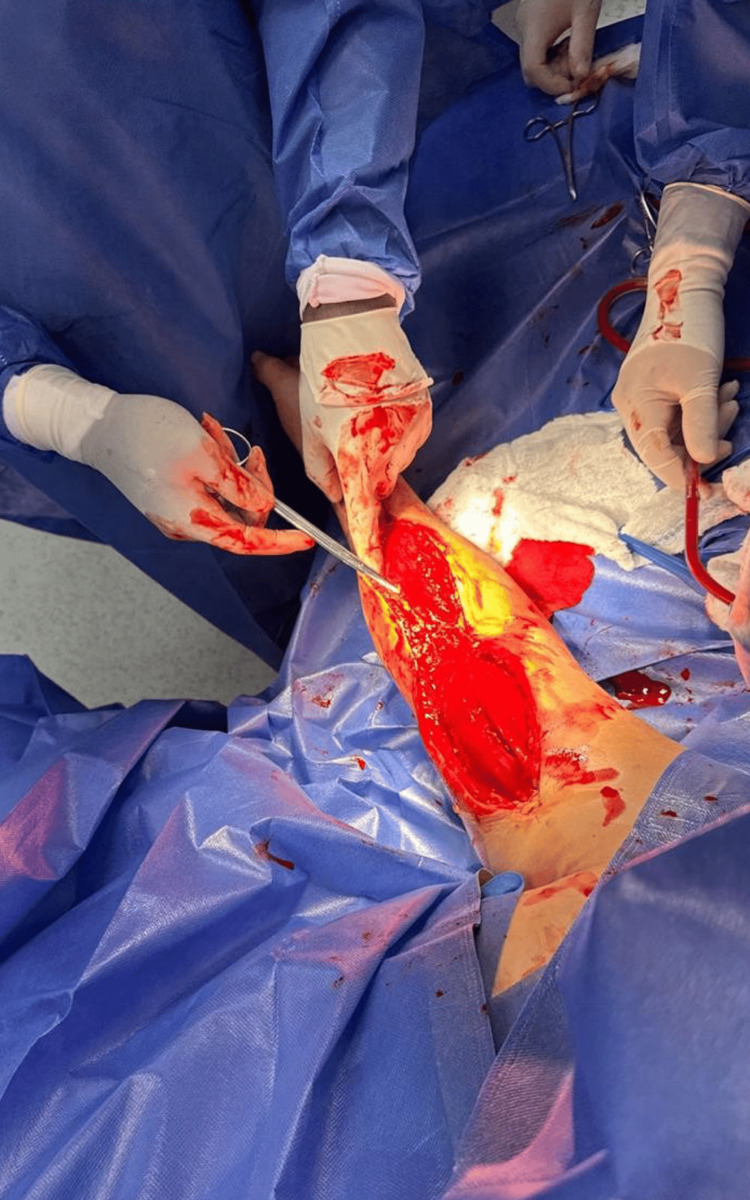
Intraoperative view of the fasciotomy procedure performed for decompression of the right upper limb compartment syndrome. The muscle compartments are exposed, demonstrating marked intramuscular edema and visible tension relief following surgical release.

Due to the patient’s hemodynamic instability, including elevated blood pressure and tachycardia, an USG-ISB was selected (Figure 3). Following the USG-ISB and surgical decompression, the patient showed measurable improvement. Within one hour, blood pressure stabilized at 122/80 mmHg, HR decreased to 88 bpm, and capillary refill improved to less than 2 seconds. The VAS pain score decreased to 3/10 during the same period. Over the next 24 hours, these parameters remained stable, with VAS pain maintained between 2 and 3 out of 10, without the need for additional opioids.

Anesthetic management

Given the clinical context and identified risks, such as an ASA physical status classification of 3E and the potential for pulmonary embolism and metabolic acidosis, the anesthesia team opted for regional anesthesia. An USG-ISB was performed using a 100×100 mm plexus block needle and a Mindray DP-50 Expert ultrasound. The anesthetic solution included bupivacaine 0.75% (60 mg), lidocaine 0.2% (80 mg), and dexamethasone 8 mg. The rationale for these doses is supported by recent literature; they are within the recommended safe ranges [6-7].

Clinical course

The immediate clinical response included a significant reduction in pain (as per the VAS), hemodynamic stabilization, capillary refill time of less than 2 seconds, and restoration of distal pulses. Local anesthetic systemic toxicity (LAST), which can cause severe cardiovascular and neurological effects, was prevented through ultrasound guidance to ensure accurate anesthetic deposition, incremental dosing with aspiration before injection, and the availability of lipid emulsion therapy in case of toxicity. Direct visualization of the needle tip and anesthetic spread under real-time ultrasound minimized the risk of nerve trauma, dissection, or vascular puncture.

By employing ultrasound guidance, careful dosing, and strict adherence to safety protocols, the risk profile of the procedure was substantially reduced. During the first 48 hours postoperatively, the patient required no opioid analgesics. Pain was effectively managed with paracetamol 1 g IV every 8 hours, confirming the expected opioid-sparing effect of the ultrasound-guided brachial plexus block (0 mg Morphine Milligram Equivalents (MME) within the first 48 hours). The block provided effective analgesia for approximately 18 hours, with VAS pain scores maintained between 2 and 3 out of 10 during this period. Mild discomfort thereafter was successfully managed with paracetamol 1 g IV every 8 hours.

**Figure 4:**
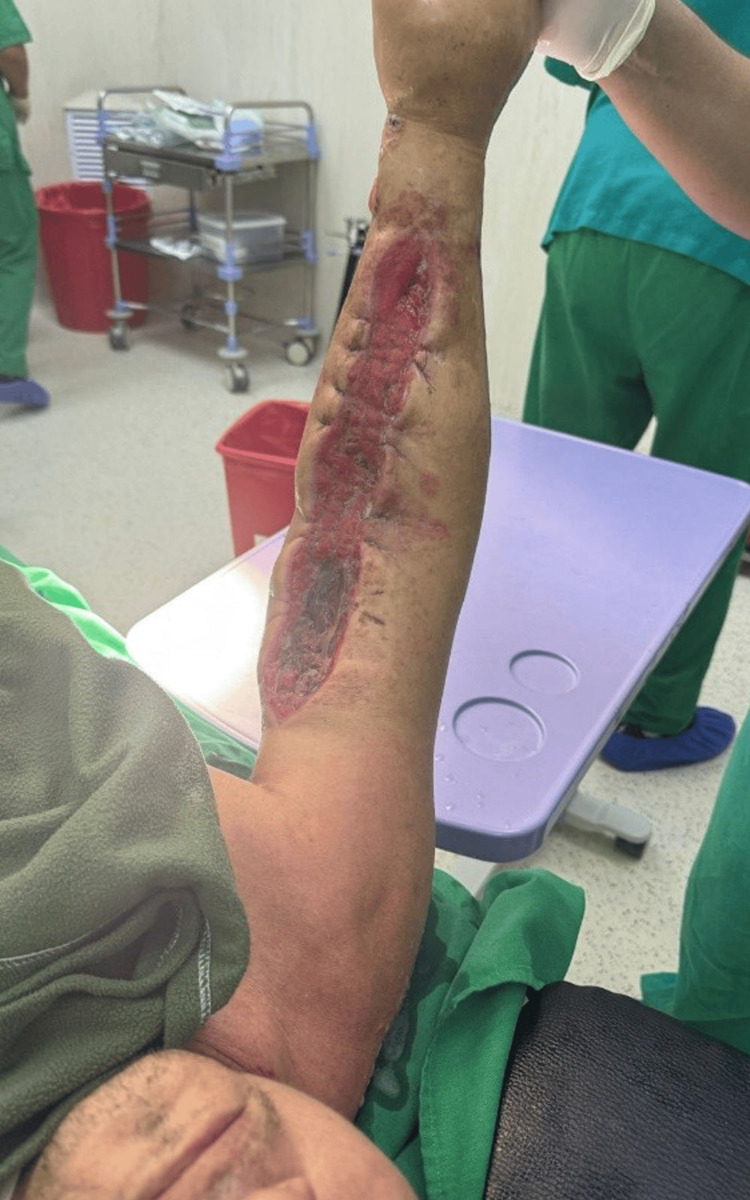
Postoperative surgical revision of the right upper limb, performed one day before hospital discharge. The fasciotomy site shows granulation tissue formation and progressive wound healing, with no evidence of infection or ischemic complications.

Throughout hospitalization, the patient remained hemodynamically stable and exhibited no neurological deficits, no clinical signs of LAST, and no recurrence of compartment syndrome. Serial neurovascular evaluations, performed hourly during the first 24 hours, showed intact motor and sensory function, normal distal perfusion, and absence of new swelling or pain. The surgical wound remained clean, without infection or delayed healing. The patient was discharged with full limb function and continued to demonstrate complete recovery without sensory changes, weakness, or ischemic sequelae. Favorable progression, absence of perioperative complications, and no subsequent morbidity were achieved after a 23-day hospital stay (Figure 4).

Monitoring protocol to detect post-block and postoperative compartment syndrome recurrence

Following the brachial plexus block and surgical decompression, the patient was placed under close and structured clinical observation to detect any recurrence of compartment syndrome. Continuous monitoring included assessment of distal pulses, limb color, temperature, capillary refill, swelling, pain level, and motor-sensory function. Evaluations were performed every 15 minutes during the first two postoperative hours, then hourly for the next 24 hours, and every four hours thereafter until clinical stabilization.

The potential masking of ischemic pain following a regional block was a major consideration. To mitigate this risk, a structured multidisciplinary monitoring protocol was implemented. The patient was kept under continuous pulse oximetry on the affected limb and closely observed in the postoperative unit. Serial neurovascular checks, including assessment of distal pulses, limb color, temperature, capillary refill, swelling, and motor-sensory function, were performed every 15 minutes during the first two postoperative hours, then hourly for the next 24 hours, and every four hours thereafter until clinical stabilization. The patient maintained stable perfusion and showed progressive recovery throughout hospitalization. The monitoring was performed collaboratively by the nursing staff, anesthesiologist, and surgical teams.

Following surgical decompression and administration of the ultrasound-guided brachial plexus block, the patient exhibited gradual and consistent clinical improvement within the first 24 hours. During the immediate postoperative period, there was a notable decrease in pain intensity, and distal perfusion improved, evidenced by the restoration of radial and ulnar pulses and a capillary refill time of less than two seconds. Over the next 6-8 hours, the edema progressively subsided, and the skin color and temperature normalized, indicating recovery of microvascular flow. The patient reported relief from the deep, pressure-like pain characteristic of compartment syndrome, though mild tingling persisted at the fingertips.

Serial neurovascular evaluations were performed hourly during the first 24 hours, documenting stable hemodynamics, preserved sensation, and no recurrence of swelling or tension. The surgical wound remained clean, with no signs of infection or secondary ischemia. By the end of the first postoperative day, the patient was comfortable, alert, and reported minimal discomfort.

Follow-up outcome

At discharge, the patient demonstrated significant clinical improvement, with complete resolution of resting pain and restoration of distal pulses. Edema was markedly reduced, and capillary refill time remained under two seconds. Sensory function in the right upper limb improved gradually, with only mild paresthesia persisting at the fingertips. Motor function was preserved, and no signs of ischemic contracture were observed.

During hospitalization (23 days), the QuickDASH score at discharge was 50/100, reflecting moderate disability, mainly affecting physically demanding tasks. At one month, the patient showed marked clinical improvement, with resolution of resting pain, reduced edema, and improved mobility. The QuickDASH score improved to 25/100, consistent with mild transient disability and minimal limitation during heavy exertion.

By the third month of follow-up, functional recovery was nearly complete. The patient exhibited a full range of motion, restoration of grip strength, and no neurological sequelae. Functionally, he was able to reintegrate into daily and occupational activities without limitation, with a QuickDASH score of 5/100, indicating minimal disability. Overall, the prognosis was favorable, underscoring the effectiveness of the combined approach of early surgical fasciotomy and ultrasound-guided regional anesthesia.

## Discussion

Regional anesthesia for the management of ULCS has been used controversially because of the associated risk of masking symptoms that are important for timely diagnosis and treatment. ULCS is a surgical emergency that requires immediate intervention, as failure to treat promptly can result in irreversible damage to muscles and nerves, leading to necrosis. Permanent tissue damage and functional impairment can occur if surgical decompression (fasciotomy) is not performed within six hours of symptom onset [8]. One of the major concerns related to the use of regional anesthesia in ULCS is that analgesia may obscure one of the key diagnostic symptoms, pain, which could result in misdiagnosis or delay in treatment.

Despite these concerns, recent literature argues that regional anesthesia does not necessarily delay the diagnosis of ULCS if proper monitoring and clinical vigilance are maintained. In fact, in some cases, it may even facilitate diagnosis by helping to differentiate ischemic pain from other pain sources. For instance, Driscoll EB et al. (2016) performed a systematic review examining the use of regional anesthesia in trauma settings, assuming adequate monitoring conditions were in place. Their results suggested that regional anesthesia can help clinicians detect pain disproportionate to the injury, which may serve as an early warning sign of compartment syndrome [9]. This observation is important because it implies that, under appropriate monitoring, regional anesthesia does not mask the diagnosis but rather may highlight disease progression by revealing abnormal pain patterns.

Supporting this reasoning, Chembrovich S et al. (2024) reinforced the concept that careful clinical observation and post-regional anesthesia patient monitoring may actually form the basis for early detection of ULCS. In cases of regional anesthesia involving sensory blockade, patients may still report pain that is disproportionate to the trauma, even before other signs of compartment syndrome become evident [10]. This vigilant monitoring approach is particularly valuable when managing high-risk patients in trauma settings.

In the present case, the patient also demonstrated clinical manifestations of sympathetic stimulation (e.g., hypertension and tachycardia), which further increased the risk of aggravation. Considering the potential hemodynamic instability associated with general anesthesia, an opioid-sparing regional anesthesia technique was selected as the preferred modality to provide effective analgesia without significant hemodynamic alterations. Bupivacaine, a long-acting local anesthetic, was therefore an appropriate choice for achieving optimal pain relief and maintaining hemodynamic stability [11,12]. By reducing catecholamine release, regional anesthesia helped interrupt the vicious cycle of pain and sympathetic hyperactivity that could otherwise lead to further ischemia and worsening of compartment syndrome.

Our case presented with high sympathetic activation (hypertension and tachycardia) and severe pain, which risked worsening the patient’s condition and potentially necessitating intermediate care. To prevent this, an ultrasound-guided regional anesthesia technique was selected because it provides effective analgesia, reduces systemic opioid exposure, and minimizes the risk of hemodynamic instability associated with general anesthesia. The long-acting agent chosen was bupivacaine [11]. Its sustained analgesic effect is due to increased lipid solubility and a high degree of protein binding, resulting in prolonged tissue retention and slow release. Regional anesthesia during the critical post-procedural stage helped dissipate the catecholamine-driven vicious cycle related to severe pain and sympathetic hyperactivity [11].

Lidocaine was also used because bupivacaine has a relatively slow onset of action. This combination allowed for effective pain management in an emergency setting, providing immediate relief while maintaining long-lasting analgesia. The concomitant use of lidocaine and bupivacaine is common in regional anesthesia to combine rapid onset (from lidocaine) with prolonged duration (from bupivacaine). The block also induces sympathetic blockade, enhancing local vascularization and potentially reducing ischemia, an especially valuable feature in compartment syndrome [12].

As an adjuvant, dexamethasone (DEX) was included. Numerous studies have shown that perineural DEX significantly prolongs both sensory and motor block durations, reduces rebound pain, and lowers the need for rescue analgesia, all without notable safety concerns [13]. This addition was beneficial for maintaining stable analgesia without systemic opioid use. Thus, this multimodal therapy optimized both the rapid onset and duration of the block, ensured effective pain control, and contributed to the patient’s overall well-being and recovery. It is noteworthy that regional blockade can also help manage mild residual pain with minimal opioid requirements and may contribute to a shorter hospital stay [14].

In this case, the authors used a mixture of bupivacaine 0.75% (60 mg), lidocaine 0.2% (80 mg), and DEX 8 mg for the posterior interscalene brachial plexus block. The dosing and volume of local anesthetics were selected to balance efficacy and safety, minimizing the risk of toxicity or complications. The 60 mg dose of bupivacaine (equivalent to 8 mL of a 0.75% solution) falls within the established safe range. Clinical guidelines state that the maximum recommended dose of bupivacaine without epinephrine is 2.5 mg/kg in adults, approximately 175 mg for a 70 kg individual. Therefore, only 34% of the maximum dose was used, consistent with safety recommendations for brachial plexus blocks [6-7]. Similarly, 80 mg of lidocaine (equivalent to 40 mL of a 0.2% solution) is well within the safe range. The maximum safe dose of lidocaine without epinephrine is 4.5 mg/kg (about 315 mg for a 70 kg adult), and the dose used in this case represents only one-fourth of that limit [6-7]. DEX (8 mg) was added to extend the duration of analgesia. This agent has been widely used as an adjuvant in regional anesthesia to enhance the quality and duration of nerve blocks, with supporting literature showing substantial reductions in postoperative pain and opioid consumption without compromising safety [15]. Together, these agents provided balanced anesthesia with a rapid onset (from lidocaine) and long-lasting analgesia (from bupivacaine), optimizing pain management after surgery.

The combination of lidocaine and bupivacaine resulted in the immediate onset of effective analgesia, an essential benefit in emergency settings. Lidocaine’s rapid onset provided early relief, while bupivacaine’s prolonged duration maintained sustained analgesia, which is particularly advantageous in trauma cases. The addition of DEX further enhanced and extended the analgesic effect, reducing the need for systemic opioids and minimizing postoperative complications [13].

Despite the advantages of regional anesthesia in this context, caution is necessary, especially in emergency trauma situations. The concern regarding masking of ULCS symptoms is justified, particularly if anesthesia is administered without adequate monitoring. The physiological response to ischemic pain can be misinterpreted when pain is obscured by regional blocks. Therefore, continuous monitoring of clinical status, pain level, and physical findings is critical for early detection and appropriate intervention [14,15].

One of the most significant benefits of ultrasound-guided regional anesthesia is its contribution to patient-centered care. Such an analgesic approach is especially valuable in trauma and emergency settings. By allowing real-time visualization of local anesthetic delivery, ultrasound guidance ensures precise administration and enhances safety. This approach reduces intraoperative pain and anxiety, supports effective surgical management (such as fasciotomy), and preserves respiratory and circulatory stability, as observed in this case [16].

ULCS has been discussed previously, and although regional anesthesia may serve as a helpful temporizing measure to maintain hemodynamic stability and pain control, definitive management requires surgical fasciotomy for direct decompression of the affected compartments and restoration of perfusion. The role of regional anesthesia should therefore be regarded as a supportive tool for analgesia and sympathetic stabilization, but not as a substitute for timely surgical intervention, which is essential to prevent irreversible tissue damage [16,17].

In conclusion, the multimodal approach combining an ultrasound-guided brachial plexus block with DEX provided safe and effective analgesia, maintained hemodynamic stability, and likely contributed to the patient’s favorable functional outcome. A QuickDASH score of 5 at the third-month follow-up reflects the excellent functional recovery achieved after fasciotomy and regional anesthetic management. These findings align with literature emphasizing the importance of early decompression and effective pain management to minimize long-term disability and facilitate optimal recovery following treatment for ULCS [18].

While the benefits of regional anesthesia in ULCS are well recognized, the potential risk of masking clinical symptoms remains a valid concern in practice. This case demonstrates that, with appropriate patient selection and vigilant monitoring under ultrasound guidance, regional anesthesia can be administered safely without delaying diagnosis. However, this must be complemented by continuous vigilance and a structured multidisciplinary approach to patient evaluation to ensure that definitive treatment (fasciotomy) is not postponed. The potential risks of regional anesthesia should be mitigated through real-time monitoring and coordinated clinical assessment by the multidisciplinary care team.

## Conclusions

The USG-ISB block is a safe and effective technique for administering regional anesthesia, contributing to hemodynamic stabilization during surgical decompression of ULCS. Surgical decompression (fasciotomy) provides definitive treatment by relieving compartment pressure, improving perfusion, and restoring blood flow, while the USG-ISB block facilitates adequate pain control and hemodynamic stability throughout the procedure. This case report demonstrates that regional anesthesia can be administered safely to achieve effective analgesia under structured monitoring and ultrasound observation without delaying the diagnosis of ULCS due to masking of cardinal symptoms. It supports timely diagnosis and intervention to preserve both life and limb, even in resource-limited settings. A favorable outcome was achieved through surgical decompression combined with effective analgesia using the USG-ISB block, resulting in optimal recovery with only mild disability (QuickDASH score of 5) at the 3-month follow-up. Overall, the prognosis was excellent, underscoring the advantages of ultrasound-guided regional anesthesia, with no perioperative complications reported.
